# COVID-19 Vaccine-Induced Myocarditis: A Systemic Review and Literature Search

**DOI:** 10.7759/cureus.27408

**Published:** 2022-07-28

**Authors:** Zahid Khan, Umesh Kumar Pabani, Amresh Gul, Syed Aun Muhammad, Yousif Yousif, Mohammed Abumedian, Ola Elmahdi, Animesh Gupta

**Affiliations:** 1 Acute Medicine, Mid and South Essex NHS Foundation Trust, Southend on Sea, GBR; 2 Cardiology and General Medicine, Barking, Havering and Redbridge University Hospitals NHS Trust, London, GBR; 3 Cardiology, Royal Free Hospital, London, GBR; 4 Internal Medicine, Barking, Havering and Redbridge University Hospitals NHS Trust, London, GBR; 5 General Practice, Starcare Hospital, Duqm, OMN; 6 Cardiology, Mid and South Essex NHS Foundation Trust, Southend on Sea, GBR; 7 Geriatrics, Barking, Havering and Redbridge University Hospitals NHS Trust, London, GBR; 8 Internal Medicine, Barking, Havering and Redbridge University Hospitals NHS Trust, Romford, GBR; 9 Acute Internal Medicine, Southend University Hospital, Southend on Sea, GBR; 10 Acute Internal Medicine and Intensive Care, Barking, Havering and Redbridge University Hospitals NHS Trust, London, GBR

**Keywords:** 12-lead ecg, pericardial diseases, cardiac chest pain, cardiac magnetic resonance imaging, cardiac troponin, post vaccination myocarditis, covid-induced myocarditis, covid 19 vaccine complication, covid vaccine-induced myocarditis, covid and myocarditis

## Abstract

Myocarditis is one of the complications reported with COVID-19 vaccines, particularly both Pfizer-BioNTech and Moderna vaccines. Most of the published data about this association come from case reports and series. Integrating the geographical data, clinical manifestations, and outcomes is therefore important in patients with myocarditis to better understand the disease. A thorough literature search was conducted in Cochrane library, PubMed, ScienceDirect, and Google Scholar for published literature till 30 March 2022. We identified 26 patients eligible from 29 studies; the data were pooled from these qualifying case reports and case series. Around 94% of patients were male in this study, the median age for onset of myocarditis was 22 years and 85% developed symptoms after the second dose. The median time of admission for patients to hospitals post-vaccination was three days and chest pain was the most common presenting symptom in these patients. Most patients had elevated troponin on admission and about 90% of patients had cardiac magnetic resonance imaging (CMR) that showed late gadolinium enhancement. All patients admitted with myocarditis were discharged home after a median stay of four days. Results from this current analysis show that post-mRNA vaccination myocarditis is mainly seen in young males after the second dose of vaccination. The pathophysiology of vaccine-induced myocarditis is not entirely clear and late gadolinium enhancement is a common finding on CMR in these patients that may indicate myocardial fibrosis or necrosis. Prognosis remains good and all patients recovered from myocarditis, however further studies are advisable to assess long-term prognosis of myocarditis.

## Introduction and background

Myocarditis is inflammation of the myocardium that can occur due to a variety of reasons, with a viral infection being the most common cause for it. Myocarditis and its related complications are believed to be largely immune-mediated [1]. The most common presentation of myocarditis is with chest pain, which can result from associated pericarditis or coronary artery spasm [2,3]. Pericarditis commonly presents with sharp, retrosternal chest pain that is exacerbated by coughing, breathing, and lying in the supine position and is relieved by sitting or leaning forward [2]. Myocarditis can affect people of all ages but typically individuals between ages 20 and 50 and diagnosis can be challenging due to the significant variation in the clinical presentation [4]. Most patients present with chest pain as the main presenting complaint (85-95% of cases), fever (65%), and dyspnoea (19-49%) of patients [4,5]. It is however important to differentiate myocarditis from acute coronary syndrome and pericarditis as they can present with similar clinical features [5]. About 80% of patients with acute myocarditis have a history of a preceding viral cold, respiratory or gastrointestinal problems [1].

Myocarditis can be classified into acute, fulminant, subacute, and chronic forms depending on the onset of symptoms [4, 6]. Acute myocarditis presents within < 1 month of onset of symptoms and diagnosis whereas fulminant myocarditis is associated with cardiogenic shock and requires inotropes or mechanical circulatory support and is a severe form of acute myocarditis that evolves rapidly. Subacute myocarditis present between one to three months after the onset of symptoms and diagnosis whereas in the case of chronic myocarditis, symptoms persist for over a month representing a kind of chronic inflammatory cardiomyopathy [4, 6].

Based on the International Classification of Disease (ICD), there were 32 cases of myocarditis per 100'000 patients reported by the Global Burden of Disease study based on the hospital discharge summaries between 1990 and 2013 [6]. The National Health Services (NHS) England data between 1998 and 2017 reported 12,1929 admissions with myocarditis which accounts for 0.04% or 36.5 per 100,000 admissions and approximately two-thirds of these patients were men and the median age was 33 years for men and 46 years for women [7]. The median length of hospital stay for both genders was 4.2 days and the number of admissions is most likely underreported. The data also showed an increasing burden of hospitalization with the disease and there was an 88% increase in admission with myocarditis over the study period compared to a 57% increase in all cardiology admissions [7]. The study also reported a higher incidence of myocarditis cases in winter and was reported as 27% in London and 16% in the Southeast even after adjusting for the regional population differences. Around 20% of mortality was due to non-ischaemic dilated cardiomyopathy and all-cause mortality was 4.16 in the last completed year of data.

COVID-19, caused by coronavirus SARS-CoV-2, led to a worldwide pandemic and a public health emergency. Prodigious immunization campaigns have been initiated throughout the world as per the World Health Organization (WHO) recommendations. This has led to the use of several types of coronavirus vaccines. These include mRNA-based vaccines, inactivated whole viral vaccines, and recombinant adenoviral vector vaccines [3,4]. The accelerated spread of the virus, as well as the unfortunate significant mortality associated with its replication, led to the emergency use of COVID-19 vaccines; the emergency approval of vaccines being against the normal standard protocol of multiple clinical trials taking place to prove the safety of the vaccines. This led to several mild to moderate and serious side effects that have been studied extensively. Few systemic reviews have been published in the past to describe the key characteristics of patients developing COVID-19 myocarditis after receiving vaccinations against the disease. It is important to mention that the most common vaccines received by patients were Pfizer, Moderna, and AstraZeneca vaccines [7,8]. A major side effect reported was myocarditis associated with these vaccines [9]. Myocarditis was more common in patients who received the COVID-19 mRNA vaccine as compared to the non-mRNA vaccine and was also higher in patients who received the second dose of the vaccine. It was also reported to be more common in males and in patients aged 16-39 years [9,10]. The aim of this systemic review is to assess various risk factors and the association of these vaccines with myocarditis.

The study is registered with PROSPERO under registration number CRD42022341932. 

## Review

Methodology

**Figure 1:**
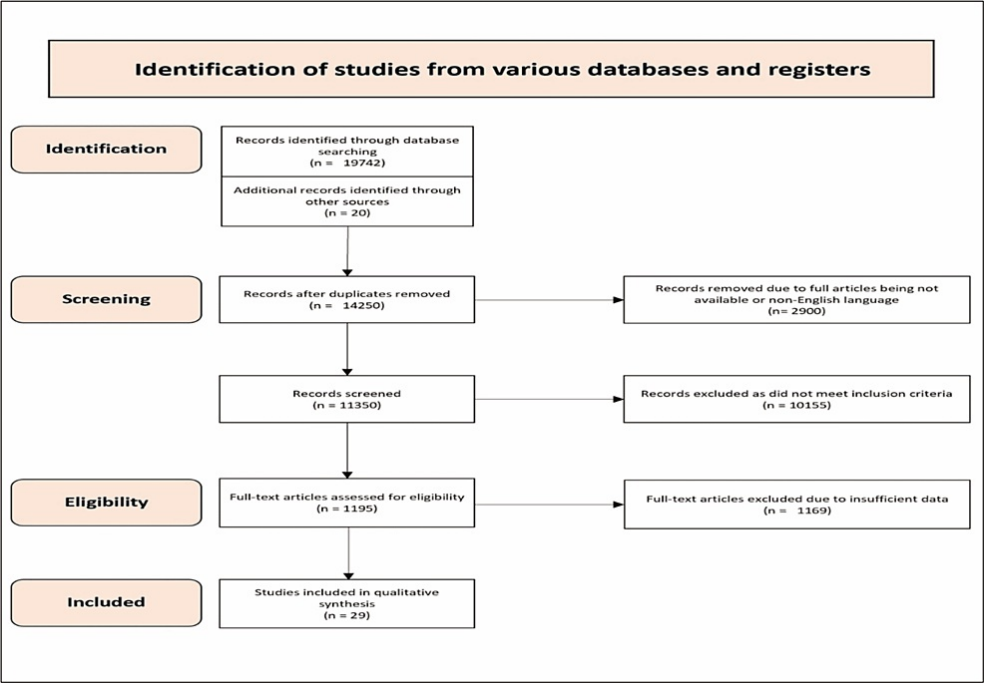
shows PRISMA 2020 flow chart for the systemic reveiw

Reporting items for systematic reviews and meta-analyses (PRISMA) protocol was used to search for studies. An electronic literature search was performed on Google Scholar, PubMed, Cochrane library and ScienceDirect. PRISMA allowed for a more systemic approach in order to identify articles for inclusion in the study. Various search terminologies were used for the literature search, including "Covid 19 vaccine and myocarditis", "COVID-19 vaccine side effects", "myocarditis, Covid 19 and myocarditis", "AstraZeneca vaccine and myocarditis", "Pfizer vaccine and myocarditis", Moderna vaccine and myocarditis", "myocarditis and pericarditis", "myopericarditis," "COVID-19 myocarditis", "vaccine-induced myocarditis", and "Covid 19 vaccine and myopericarditis". The article search was undertaken in April 2022 and a total of 19,742 articles were found through a structured search. An additional 20 articles were found through a Google search. A total of 11,350 articles were left after removing duplicate articles and articles that were not available or were not in the English language. A further 10,155 articles were removed for not meeting the inclusion criteria. As a result, 1,195 articles were screened for eligibility and 1,169 articles were removed for not providing enough information. Finally, 29 articles were included in the systemic review as shown in the PRISMA chart (Figure 1) which were relevant to our study.

Table 1 shows the regular and MeSH keywords used for the literature search.

**Table 1:** shows regular and mesh key words used for literature search

Search	Keywords
Regular Keywords	Covid 19 vaccine, Myocarditis, Myopericarditis, covid 19 vaccine side effects, Pfizer vaccine, Moderna vaccine, AstraZeneca vaccine, myopericarditis, pericarditis
MeSH keywords	Covid 19 vaccine and myocarditis, COVID-19 vaccine side effects, myocarditis, Covid 19 and myocarditis, AstraZeneca vaccine and myocarditis, Pfizer vaccine and myocarditis, Moderna vaccina and myocarditis, myocarditis, and pericarditis, myopericarditis, COVID-19 myocarditis, vaccine induced myocarditis, Covid 19 vaccine and myopericarditis”.

Methods

Eligibility Criteria 

Inclusion and exclusion criteria were determined and accordingly, articles were assessed. Approximately 50 studies met the criteria, however, after removing duplicate articles, only 29 articles were finally included in the systemic review. The inclusion and exclusion criteria are described below.

Inclusion Criteria

The inclusion criteria were: (1) Patients should have received the COVID-19 vaccination within the last two months; (2) patients should have elevated troponin and echocardiographic/cardiac magnetic resonance imaging findings suggestive of myocarditis; (3) patients should not have had a COVID-19 infection in the preceding month; (4) articles should be written in English; (5) articles should be available through institutional access or be free; and (6), patients should be older than 14 years old.

Exclusion Criteria

The exclusion criteria were: 1 - Studies in which patients did not have the COVID-19 vaccine within the last two months prior to developing Myocarditis. 2 - Studies in which patients had the COVID-19 vaccine within one month of having a COVID-19 infection. 3 - Studies in which patients did not have an echocardiogram, troponin, coronary angiogram +/- CMR to confirm the diagnosis of myocarditis. 4 - Studies in which patients were under 14 years of age. 5 - Studies in which patients had a diagnosis of myopericarditis. 6 - Articles that were not in English and were not accessible through institutional access or were not free.

Study Selection and Data Extraction

All peer-reviewed published studies that included patients above the age of 14 who developed myocarditis following any type of COVID-19 vaccine (mRNA, viral vector, and protein subunit) were included. Review articles, editorials, preprints, and original articles that reported side effects of vaccination but did not discuss myocarditis specifically were excluded.

Data Collection Process and Data Items

The data were extracted independently by two authors using standardized data extraction forms. Data points collected included age, gender, type of vaccination, clinical features, number of days following a vaccine that the symptoms occurred, outcome, laboratory values, methods of diagnosis, and results on an Excel sheet (Microsoft Corporation, Redmond, WA).

Study Analysis

Patient demographic characteristics, disease manifestations, and causes were summarized descriptively.

Results

The results of 29 studies are described below in Table 2. The summaries are pooled together in Table 3.

**Table 2:** Results from 29 studies The table shows results from 29 studies outlining sex, age, number of days between vaccination and admission to hospital, ECG results, cardiac MRI results, use of anti-inflammatory medication, and past medical history LGE: late gadolinium enhancement; AV block: atrioventricular block; VT: ventricular tachycardia, RBBB: right bundle branch block, LV: left ventricle; RV: right ventricle

Author	No. of patients	Male (%)	Age (years)	Hospital presentation (days after vaccination)	Symptoms reported	EKG changes	MRI findings	Anti-inflammatory treatment used	Previous comorbidities
Montgomery et al. 2021 [11]	23	100	25	4	Chest pain at rest	ST-segment elevations, or T-wave inversions, non-specific T waves and ST-segment changes.	Subepicardial LGE and/or focal myocardial oedema	No	No
Garcia et al. 2021 [12]	1	100	39	1	Intermittent chest and interscapular pain	Sinus tachycardia, narrow QRS complex, diffuse ST-elevation	Oedema on T2-weighted short-tau inversion recovery sequences and subepicardial enhancement in the lateral mediastinal region	Yes	Asthma, atrial fibrillation and hypothyroidism
Kim et al. 2021 [13]	7	85	23	5	Severe chest pain	Abnormal (not described)	Regional wall motion abnormalities, evidence of LGE, and elevated native T1 and T2	No	No
Shaw et al. 2021 [14]	3	67	24	4	Chest pain	ST-elevation	Epicardial oedema, epicardial fibrosis, regional interstitial expansion	No	No
Jain et al. 2021 [15]	63	92	16	2	Chest pain, fever and nausea	Diffuse ST-elevation, T-wave inversion	Myocardial injury as evidenced by LGE	No	No
Truong et al. 2021 [16]	139	91	16	2	Chest pain	Diffuse ST-elevation, non-sustained VT	LGE, myocardial oedema	Yes	No
D'Angelo et al. 2021 [17]	1	100	30	3	Chest pain, nausea, profuse sweating	subtle ST-segment elevation suggestive of potential myocardial injury or pericarditis in V2-V4 and nonspecific T-wave changes in V5 and V6	Subepicardial enhancement of the myocardium, enhancement of pericardium was also seen	Yes	No
Perez et al. 2021 [18]	7	86	25	3	Chest pain, dyspnoea and fatigue	ST-segment changes	Myocardial delayed enhancement	Yes	Hypertension, obesity, obstructive sleep apnea, smoking and dyslipidemias
Muthukumar et al. 2021 [19]	1	100	52	3	Chest pain	Sinus rhythm with left axis deviation and incomplete right bundle-branch block without ST- or T-wave changes	Midmyocardial and subepicardial linear and nodular LGE in the inferoseptal, inferolateral, anterolateral, and apical walls	Yes	Hypertension, hypercholesterolemia, obstructive sleep apnea
Nevet et al. 2021 [20]	3	100	24	2	Chest pain	Diffuse ST-elevations	Myocardial oedema and gadolinium enhancement of the myocardium	Yes	No
Naghashzadeh et al. 2022 [21]	1	100	29	2	Chest pain	ST-segment elevation	Not done acutely	Yes	Yes
Gautam et al. 2021 [22]	1	100	66	90	Chest pain and diaphoresis	1 mm ST-elevation on anterior leads.	Moderately impaired left ventricular systolic function with LV ejection fraction of 44%, presence of myocardial and epicardial enhancement at a mid-ventricular level along the anterior septum extending to base, sparing the subendocardium	Yes	Hypertension, type II diabetes mellitus, and hyperlipidemia
Parmar et al. 2021 [23]	4	75	22	3	Chest pain, tachycardia	AV block, diffuse ST-elevation	Mild LGE is seen in the inferolateral region in the pericardium	Yes	No
Watkins et al. 2021 [24]	1	100	20	2	Chest pain and mild shortness of breathing	diffuse concave ST-segment elevations with PR depressions	Positive for myocarditis; details not included	Yes	No
Łaźniak-Pfajfer et al. 2021 [25]	3	100	17	2	Chest pain	Negative T-waves in the inferior leads and flat T waves in V6 in one of the patients	LGE, pericardial effusion	No	No
King et al. 2021 [26]	4	100	23	4	Chest pain	Down-sloping PR depressions and diffuse ST-elevations	Delayed gadolinium enhancement suggestive of fibrosis involving the mid to apical anterolateral wall segments	No	No
Fosch et al. 2022 [27]	1	100	24	1	Chest pain and fever	Concave ST-elevation	Oedema in basal. LGE showed patchy, subepicardial enhancement	Yes	Yes
Schmitt et al. 2021 [28]	1	100	19	3	Chest pain	Persistent ST-elevation without reciprocal depression	LGE sequences identifying a lateral subepicardial enhancement	No	No
Shumkova et al. 2021 [29]	1	100	23	1	Chest pain, shortness of breathing and fever	ST-elevation in inferior and V4-V6	T2-weighted images showed increased signal intensity in basal segments indicating interstitial oedema	Yes	No
Cui et al. 2021 [30]	2	50	57	4	Chest tightness, fever, chills, tiredness and chest pain	RBBB, ST-elevation on anterior leads with third-degree atrioventricular block	LGE imaging demonstrates myocardial necrosis in the middle ventricular septum with thinning of the lateral wall and formation of fibrosis, myocardial oedema	Yes	No
Azir et al. 2021 [31]	1	100	17	3	Chest pain and fever	Sub-1-mm lateral ST elevations with sub-1-mm depression in lead III	Diffuse, subepicardial delayed gadolinium enhancement of the anterior and lateral wall of the left ventricle, with corresponding heterogeneous T1 signal prolongation and increased short tau inversion recovery signal	Yes	No
Mansour et al. 2021 [32]	2	50	21	1	Chest pain and fever	Mild diffuse concave ST elevation without reciprocal changes	Subepicardial enhancement in the inferolateral wall at the base	No	No
Riedel et al. 2021 [33]	1	100	47	14	Chest pain, fever and associated pneumonia attacks	Sinus tachycardia and left ventricular overload	Hypokinetic LV and RV in cardiac MRI, biatrial dilation, mitral and tricuspid insufficiency, and late enhancement of non-ischemic aspect	No	type II diabetes
Sciaccaluga et al. 2022 [34]	2	100	20	3	Fever and chest pain	Sinus rhythm, normal atrioventricular conduction, incomplete right bundle branch block	Myocardial oedema and LGE with subepicardial pattern	Yes	No
Murakami et al. 2022 [35]	2	100	30	5	Chest pain	ST-elevation in multiple leads	LGE showed a subepicardial lesion in anterolateral segments at the left ventricular mid-apical level	Yes	No
Kerkhove et al. 2022 [36]	1	100	50	5	Shortness of breathing, malaise and fever	Not provided	Belated contrast capitation in the left ventricle	No	No
Tailor et al. 2021 [37]	1	100	44	4	Chest pain	ST-segment elevation in the lateral limb and precordial leads	Linear mid-myocardial LGE of the septum and inferior walls at the base to mid-ventricle, sub-epicardial/mid-myocardial enhancement of the apical lateral wall	Yes	Yes
Ohnishi et al. 2021 [38]	1	100	26	1	Chest pain, fever and malaise	ST-elevation with upward concavity in I, II, aVL, aVF, V4 to V6, and small Q wave in II, III, aVF	Oedema and LGE of the left ventricle in a mid-myocardial and epicardial distribution	Yes	No
Kawakami et al. 2022 [39]	1	0	45	7	Fever and chest pain	T-wave inversion in inferior leads on Day 1, V5-V6 on Day 2, normalized on Day 7	LGE demonstrated diffuse hyperenhancement, especially in the apex, inferior and lateral walls	Yes	No

**Table 3:** Summary of pooled data from all 29 studies CRP: C-reactive protein; BNP: brain natriuretic peptide; ESR: erythrocyte sedimentation rate; LAD: left axis deviation:  RBBB: right bundle branch block; EF: ejection fraction; LGE: late gadolinium enhancement; NSAIDS: non-steroidal anti-inflammatory drugs

Summary of pooled data from included studies	
Age	Total patients - 276
	Median - 22 years (range 17–66 years)
Sex (n)%	Male - 262 (94%)
	Female - 14 (6%)
Symptom onset after vaccination	Median - 3 days (range--> 1–30 days)
	After first dose (n=2): median 4 days (range--> 2–25 days)
	After second dose (n=262): median 3 days (range -->0–4 days)
Days to hospitalization after vaccination	Median - 3 days (range--> 1–25 days)
Symptoms (n)%	Chest pain/tightness - 276 (100%)
	Fever - 170 (62%)
	Myalgia/generalized body ache - 16 (6%)
	Chills/rigors - 15 (5%)
	Dyspnea/sob - 13 (5%)
	Fatigue - 8 (3%)
Highest reported value of troponin	Troponin I (24) - median--> 8.161 ng/mL (range --> 0.37–44.8 ng/mL)
	Troponin T (19) - median--> 1.332 ng/mL (range -->0.39– 3.72 ng/mL)
	High sensitivity troponin T (9) - median--> 0.70 ng/mL (range --> 0.18–15.34 ng/mL)
	High sensitivity troponin I (4) - median -->6.90 ng/mL (range--> 6.77–14.35 ng/mL)
	Troponin reported as multiple of upper limit of normal (8). Median --> 192.5 (range --> 29–1433).
	Troponin not specified (1) - value 0.11 ng/mL
	High-sensitivity troponin not specified (1) - value 32.14 ng/mL
	Reported to be elevated (3)
Elevated inflammatory and other cardiac biomarkers (n)%	Reported - 152/276 patients (55%)
	CRP - 48 (18%)
	ESR - 15 (6%)
	BNP/Pro-BNP - 18 (7%)
EKG (n)%	Reported - 275/276 patients (99.9%)
	ST-elevation - 260 (94%)
	PR depression - 25 (9%)
	ST-depression - 30 (11%)
	Peaked T-waves - 5 (1.8%)
	LAD with partial/incomplete RBBB - 25 (9%)
Echocardiography (n)%	Reported - 155/276 (57%)
	EF > 50% with no regional wall abnormalities - 137 (49.6 %)
	Hypokinesis - 18 (7%)
	EF < 50% - 25 (9%)
	Pericardial effusion - 27 (7.2%)
Cardiac MRI (n)%	Reported - 276/276 patients (100%)
	Positive for myocarditis - 260/276 (89.1%)
	LGE reported - 210 (76%)
Treatment (n)%	Specific anti-inflammatory therapy reported in 158/276 patients (57.2%)
	NSAIDS - 158 (57.2%)
	Colchicine - 121 (43.8%)
	Steroids - 69 (25%)
Recovery	Reported 276/276 patients (100%)
	100% recovery rate

Most of the patients who presented with chest pain were later diagnosed with myocarditis. Out of 276 patients, the mean age was 22 years, ranging from ages 17 to 66. Quite significantly, the vast majority of patients who developed myocarditis were male - 262 out of 276 (94%) - suggesting that the male population was more susceptible to having myocarditis. Additionally, the majority of patients (262) developed myocarditis symptoms after the second dose of their COVID-19 vaccination. The median number of days between vaccination and admission to hospital was three days. In terms of symptoms, all 276 patients developed chest pain (100%). More than half had fevers - 170 (62%). Other symptoms reported were chills (5%), dyspnoea (5%), and fatigue (3%).

Most patients had ECG abnormalities and about 260 (94%) patients had ST-segment elevation. Most patients had elevated inflammatory markers such as troponin I, C-reactive protein (CRP), and brain natriuretic peptide (BNP) levels. The peak troponin-I level was 162.275 ng/ml ±754.804 ng/mL. CRP level was 26.43 mg/L ±31.98 mg/L and the BNP level was 51.31 pg/ml ± 25.64 pg/ml (Table 2).

COVID-19 PCR tests were negative in all these patients. Most patients had echocardiography and the estimated ejection fraction on echocardiography was about 51% of these patients. Only 22 patients underwent coronary angiography following ECG and echocardiography. Of these, 20 patients had completely normal coronary arteries and only two patients had mild coronary artery irregularities. Most patients had the diagnosis of myocarditis confirmed with cardiac MRI, which showed cardiac wall oedema on gadolinium enhancement with signs of hyperaemia or fibrosis. 32 patients (80%) from a total of 40 patients had myocardial wall oedema on gadolinium enhancement on CMR scans. 

**Figure 2:**
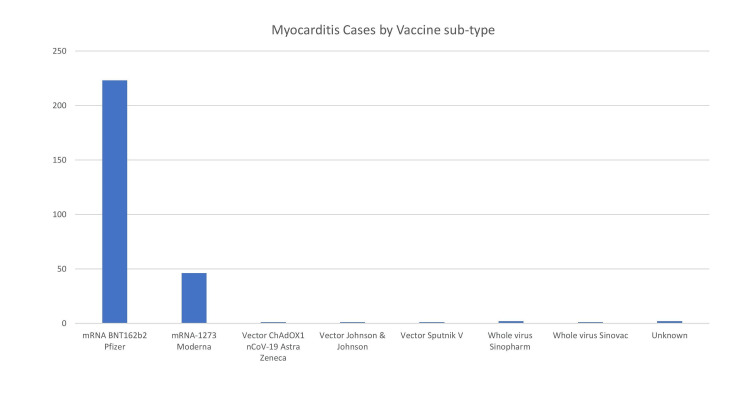
Myocarditis cases by vaccine sub-type

**Figure 3:**
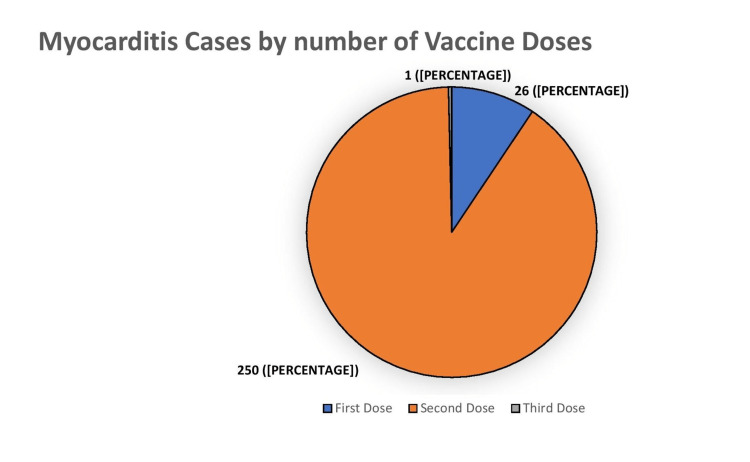
Myocarditis cases by the number of vaccine doses

The association between the type of vaccine and the dose of vaccine administered for individual studies is shown in Table 4. Most COVID-19 vaccine-induced myocarditis cases were reported with the Pfizer vaccine, followed by the Moderna vaccine (Figure 2 and Table 5). Figure 3 and Table 6 show the incidence of COVID 19 myocarditis with the number of vaccine doses and 250 cases were reported after the second dose of COVID 19 vaccination. 

**Table 4:** Incidence of myocarditis and its relation to COVID-19 vaccine dosage

Serial number	Study author	Type of vaccine	Myocarditis after first vaccine dose	Myocarditis after second vaccine dose	Myocarditis after third vaccine dose
1	Montgomery et al. 2021 [11]	mRNA BNT162b2 Pfizer 7 (30%) mRNA-1273 Moderna 16 (70%)	3 (13%)	20 (87%)	0 (0%)
2	Garcia et al. 2021 [12]	mRNA BNT162b2 Pfizer 1 (100%)	0 (0%)	1 (100%)	0 (0%)
3	Kim et al. 2021 [13]	mRNA-1273 Moderna 2 (50%) mRNA BNT162b2 Pfizer 2 (50%)	0 (0%)	4 (100%)	0 (0%)
4	Shaw et al. 2021 [14]	mRNA BNT162b2 Pfizer 3 (75%) mRNA-1273 Moderna 1(25%)	2 (50%)	2 (50%)	0 (0%)
5	Jain et al. 2021 [15]	mRNA BNT162b2 Pfizer 59 (94%) mRNA-1273 Moderna 4 (6%)	1 (1.6%)	62 (98.4%)	0 (0%)
6	Truong et al. 2021 [16]	mRNA BNT162b2 Pfizer 131 (94.2%) mRNA-1273 Moderna 5 (3.6%) Johnson & Johnson 1 (0.7%) Unknown 2 (1.4%)	12 (8.6%)	128 (91.4%)	0 (0%)
7	D'Angelo et al. 2021 [17]	mRNA BNT162b2 Pfizer 1 (100%)	0 (0%)	1 (100%)	0 (0%)
8	Perez et al. 2021 [18]	mRNA BNT162b2 Pfizer 3 (42%) mRNA-1273 Moderna 4 (57%)	1 (14.3%)	6 (85.7%)	0 (0%)
9	Muthukumar et al. 2021 [19]	mRNA-1273 Moderna 1 (100%)	0 (0%)	1 (100%)	0 (0%)
10	Nevet et al. 2021 [20]	mRNA BNT162b2 Pfizer 3 (100%)	0 (0%)	3 (100%)	0 (0%)
11	Naghashzadeh et al. 2022 [21]	rAd26 and rAd5 vector-based Sputnik V 1 (100%)	0 (0%)	1 (100%)	0 (0%)
12	Gautam et al. 2021 [22]	mRNA BNT162b2 Pfizer 1 (100%)	0 (0%)	1 (100%)	0 (0%)
13	Parmar et al. 2021 [23]	mRNA-1273 Moderna 4 (100%)	1 (25%)	3 (75%)	0 (0%)
14	Watkins et al. 2021 [24]	mRNA BNT162b2 Pfizer 1 (100%)	0 (0%)	1 (100%)	0 (0%)
15	Łaźniak-Pfajfer et al. 2021 [25]	mRNA BNT162b2 Pfizer 3 (100%)	2 (66.6%)	1 (33.3%)	0 (0%)
16	King et al. 2021 [26]	mRNA-1273 Moderna 3 (75%) mRNA BNT162b2 Pfizer 1 (25%)	0 (0%)	4 (100%)	0 (0%)
17	Fosch et al. 2022 [27]	mRNA BNT162b2 Pfizer 1 (100%)	0 (0%	0 (0%)	1 (100%)
18	Schmitt et al. 2021 [28]	mRNA BNT162b2 Pfizer 1 (100%)	0 (0%)	1 (100%)	0 (0%)
19	Shumkova et al. 2021 [29]	mRNA BNT162b2 Pfizer 1 (100%)	1 (100%)	0 (0%)	0 (0%)
20	Cui et al. 2021 [30]	Sinopharm Vero-Cell 2 (100%)	2 (100%)	0 (0%)	0 (0%)
21	Azir et al. 2021 [31]	mRNA BNT162b2 Pfizer 1 (100%)	0 (0%)	1 (100%)	0 (0%)
22	Mansour et al. 2021 [32]	mRNA-1273 Moderna 2 (100%)	0 (0%)	2 (100%)	0 (0%)
23	Riedel et al. 2021 [33]	Sinovac 1 (100%)	0 (0%)	1 (100%)	0 (0%)
24	Sciaccaluga et al. 2022 [34]	mRNA-1273 Moderna 2 (100%)	0 (0%)	2 (100%)	0 (0%)
25	Murakami et al. 2022 [35]	mRNA BNT162b2 Pfizer 2 (100%)	1 (50%	1 (50%)	0 (0%)
26	Kerkhove et al. 2022 [36]	ChAdOX1 nCoV-19 Astra Zeneca 1 (100%)	0 (0%)	1 (100%)	0 (0%)
27	Tailor et al. 2021 [37]	mRNA-1273 Moderna 1 (100%)	0 (0%)	1 (100%)	0 (0%)
28	Ohnishi et al. 2021 [38]	mRNA BNT162b2 Pfizer 1 (100%)	0 (0%)	1 (100%)	0 (0%)
29	Kawakami et al. 2022 [39]	mRNA-1273 Moderna 1 (100%)	0 (0%)	1 (100%)	0 (0%)

**Table 5:** Myocarditis cases by the number of vaccine doses

	mRNA BNT162b2 Pfizer	mRNA-1273 Moderna	Vector ChAdOX1 nCoV-19 Astra Zeneca	Vector Johnson & Johnson	Vector Sputnik V	Whole virus Sinopharm	Whole virus Sinovac	Unknown
Cases	223	46	1	1	1	2	1	2

**Table 6:** COVID-19 vaccine-induced myocarditis cases based on vaccine doses

	First dose	Second dose	Third dose
Myocarditis cases	26 (9.4%)	250 (90%)	1 (0.4%)

In terms of treatment options, 36 patients (13%) received NSAIDs, 21 patients (8%) received colchicine, nine patients (3%) received steroids and six patients (2%) received IVIG. There was no mortality reported in all 276 patients who were all discharged home. 

Discussion

COVID-19 vaccine-related myocarditis has been reported for the past two years, mainly in case reports and only a few systemic reviews have been undertaken on this topic [40-42]. This was more commonly seen in younger males after the second dose of the mRNA vaccine. Myocarditis presentations can vary and patients can present with a range of symptoms from asymptomatic to heart failure requiring a heart transplant, or lethal heart arrhythmias and sudden cardiac death in most severe cases. Fortunately, most myocarditis cases associated with mRNA vaccines are mild in nature and do not have serious complications and require only a few days of hospital admission. According to the US Centre for Disease Control director Dr Rochelle Walensky, if one million children are fully immunized against COVID-19, 30-40 children may get mild myocarditis, however, this will prevent 8000 cases of COVID-19, 200 hospital admissions, 50 intensive care unit (ICU) stays and one death in this age group [43].

One major concerning complication of myocarditis is heart failure which could be heart failure with reduced ejection fraction (HFrEF) or heart failure with preserved ejection fraction (HFpEF). Clinically, myocarditis has three possible phenotypes which include acute myocarditis, fulminant myocarditis, and chronic active myocarditis [44,45]. Acute myocarditis patients usually have mild symptoms which occur following a gastrointestinal or upper respiratory tract infection and patients have complete recovery in most cases. According to the Marburg Myocarditis Registry, 2.5% of patients out of 1000 biopsy-confirmed myocarditis patients presented with the fulminant phenotype; however, this has been reported to be up to 30% in other studies [46]. These patients usually have more severe symptoms compared to acute myocarditis and may require vasopressor support due to severe myocardial inflammation. It is more common in patients with underlying autoimmune conditions such as systemic lupus erythematosus (SLE), scleroderma, Sjogren’s, or those on immunosuppressive therapy [39]. Based on histology, this type of myocarditis may be lymphocytic, eosinophilic, or giant cell and an immune checkpoint inhibitor (ICI)-induced myocarditis is usually of fulminant type. The final type of myocarditis is chronic active myocarditis in which there is constant low-grade inflammation of the myocardium after initial acute myocarditis and usually leads to intramural and/or epicardial scarring progressing to dilated cardiomyopathy (DCM) [47].

The vast majority of patients who developed myocarditis after vaccination were males. This is consistent with another study done retrospectively of 40 COVID-19 vaccine-related myocarditis patients, out of whom 90% were male [48]. Additionally, the vast majority of patients who developed myocarditis did so after the second dose of their vaccine. This suggests the likelihood that the myocarditis could be "hypersensitivity myocarditis", which is a delayed, type IV type, drug-induced reaction with eosinophilic inflammation and a T helper cell type 2 response [49], with the first dose acting as a sensitizing exposure to the immunogenic trigger.

The underlying pathophysiology of vaccine-induced myocarditis remains unclear. One possible mechanism is molecular mimicry; the cardiac self-antigens being similar in structure to the spike protein of SARS-CoV-2 and so being targeted by the individual’s own antibodies, leading to cardiac inflammation [50]. These individuals might be more prone to such a response due to their own immunogenetic background, leading to hyperimmunity following vaccination. Another theory, driven by the fact that males seem to be more susceptible to myocarditis following coronavirus vaccination than women, is that testosterone and oestrogen levels mitigate an individual’s immune response. Oestrogen inhibits pro-inflammatory T cells, leading to a "softer" immune response while testosterone promotes T helper 1 cell-type response, leading to hyperimmunity [49,50].

The majority of patients diagnosed with myocarditis underwent cardiac MRI, which is the gold standard imaging for the diagnosis of myocarditis according to the European Society of Cardiology [51]. Not only does it provide accurate information about the degree of inflammation, cardiac function and viability, it is also effective in determining prognosis [51,52]. Additionally, it is an excellent imaging modality in patients who have non-diagnostic echocardiograms due to limitations such as body habitus. The management of myocarditis included non-steroidal anti-inflammatory drugs (NSAIDs), steroids, intravenous immunoglobulin (IVIG) and colchicine. Although there are no randomized clinical trials to establish the best treatment choice for vaccine-induced myocarditis [53], it is prudent to manage the condition based on its severity. For example, NSAIDs may relieve chest pain but might exacerbate heart failure through sodium retention, whereas steroids or IVIG might be more strongly indicated in patients with myocarditis-induced arrhythmias.

It is important to mention that the COVID-19 infection is also associated with myocarditis and the associated risk is higher than the mRNA vaccine-associated risk. Some studies note a mortality rate of 14% in patients with COVID-19 infection-induced myocarditis [54]. It is important to note such statistics when discussing the risks vs benefits of vaccination with patients, in order to help them make informed decisions. Table 7 shows the data for the total number of various COVID-19 vaccines administered and their reported side effects across the European Union and the European economic area (EEA) [55]. In a systemic review by Matta et al., about 93% of patients were male and the median age of onset was 21 years and almost 89% of patients developed myocarditis after the second dose [56].

**Table 7:** Total doses of various COVID-19 vaccines administered in EU and EEA and reported side effects EU: European Union; EEA: European Economic Area [56].

Vaccine type	Total number of vaccines administered	Reported side effects
BioNTech and Pfizer	627,000,000	743,735
AstraZeneca	69,000,000	276,697
Moderna	155,000,000	206,920
Janssen	19,400,000	48,410
Novavax	178,000	294

Limitations

Most articles reviewed in this systematic review were case series comprising multiple case reports of myocarditis following vaccination against coronavirus. Long-term studies that investigate the prognosis of patients who develop myocarditis need to be conducted. Additionally, the studies featured in this systematic review have highlighted cases that have been severe enough to present to medical services, whereas cases of myocarditis that have been milder or patients who have not sought medical attention have not been included meaning that there may be under-representation of myocarditis cases following coronavirus vaccination. This includes patients in developing countries who might not have the means for investigations such as cardiac MRI and might be misdiagnosed as simply having heart failure or ischaemic heart disease. This systematic review has been appraised using the Critical Appraisal Skills Programme (CASP) tool present in the appendix section [57].

## Conclusions

Post-vaccination myocarditis is predominantly seen in young male patients in their early 20,s with an average age of 21 years. Most patients with vaccine-induced myocarditis present within a few days following the second dose of COVID-19 vaccines. The most common presenting symptom is chest pain followed by fever and myalgia or general body ache. The electrocardiogram is abnormal in most patients and may show either ST-segment elevation or T waves inversion or non-specific ST-segment changes. Most patients have elevated troponin I and raised inflammatory markers on blood tests and cardiac magnetic resonance imaging shows late gadolinium enhancement which indicate myocardial necrosis or fibrosis in these patients. The diagnosis of vaccine-induced myocarditis is initially made based on the presenting history of chest pain following recent administration of mRNA vaccination, elevated troponin, abnormal electrocardiogram findings and characteristic findings on cardiac MRI. Although the prognosis is good as all the reported patients recovered and were discharged home, further research is needed to understand the pathophysiology of post-vaccination myocarditis and to improve the standard of care for these patients.

## Appendices

This review has been appraised using the Critical Appraisal Skills Programme (CASP) tool [57] (Table 8). 

**Table 8:** Critical Appraisal Skills Programme tool

Section A: Are the results of the review valid?				
Did the review address a clearly focused question?	Yes	✓		HINT: An issue can be ‘focused’ In terms of • the population studied • the intervention given • the outcome considered
	Can’t Tell			
	No			
Comments: Sufficient evidence to suggest that there is a temporal relationship between COVID-19 vaccines and myocarditis				
Did the authors look for the right type of papers?	Yes	✓		HINT: ‘The best sort of studies’ would address the review’s question have an appropriate study design (usually RCTs for papers evaluating interventions)
	Can’t Tell			
	No			
Comments: No RCT’s performed due to the nature of myocarditis occurring as a side-effect of COVID-19 vaccinations, which is why case series and reports were predominantly used				
Is it worth continuing?				
Do you think all the important, relevant studies were included?	Yes			HINT: Look for • which bibliographic databases were used • follow up from reference lists • personal contact with experts • unpublished as well as published studies • non-English language studies
	Can’t Tell	✓		
	No			
		Comments: Non-English studies were not used				
4. Did the review’s authors do enough to assess quality of the included studies?	Yes		✓	HINT: The authors need to consider the rigour of the studies they have identified. Lack of rigour may affect the studies’ results (“All that glisters is not gold” Merchant of Venice – Act II Scene 7)
	Can’t Tell			
	No			
Comments: Inclusion and Exclusion criteria were used – only studies in which Covid-19 vaccines were administered and myocarditis was definitively diagnosed were used				
5. If the results of the review have been combined, was it reasonable to do so?	Yes		✓	HINT: Consider whether • results were similar from study to study • results of all the included studies are clearly displayed • results of different studies are similar • reasons for any variations in results are discussed
	Can’t Tell			
	No			
			Comments:				
Section B: What are the results?				
6. What are the overall results of the review?				HINT: Consider • If you are clear about the review’s ‘bottom line’ results • what these are (numerically if appropriate) • how were the results expressed (NNT, odds ratio etc.)
Comments: There is a temporal relationship between Covid-19 vaccines and myocarditis – it is quite likely that Covid-19 vaccination causes myocarditis, although further studies can be done to prove this				
7. How precise are the results?				HINT: Look at the confidence intervals, if given
Comments: Due to the lack of RCT’s performed on this subject matter, it is difficult to comment on exact precision				
Section C: Will the results help locally?				
8. Can the results be applied to the local population?	Yes		✓	HINT: Consider whether the patients covered by the review could be sufficiently different to your population to cause concern your local setting is likely to differ much from that of the review
	Can’t Tell			
	No			
Comments: Same vaccines are used in local vaccination program that were used in the studies included in the systematic review				
9. Were all important outcomes considered?	Yes			HINT: Consider whether there is other information you would like to have seen
	Can’t Tell		✓	
	No			
Comments: Long-term studies on these patients are yet to be done to see the long-term effects of myocarditis				
10. Are the benefits worth the harms and costs?	Yes		✓	HINT: Consider even if this is not addressed by the review, what do you think?
	Can’t Tell			
	No			
Comments: Covid-19 itself causes myocarditis to a higher degree than vaccination does. Hence vaccination outweighs the harms and costs				

## References

[1] Lampejo T, Durkin SM, Bhatt N, Guttmann O (2021). Acute myocarditis: aetiology, diagnosis and management. Clin Med (Lond).

[2] Imazio M, Gaita F, LeWinter M (2015). Evaluation and treatment of pericarditis: a systematic review. JAMA.

[3] Checcucci E, Piramide F, Pecoraro A (2022). The vaccine journey for COVID-19: a comprehensive systematic review of current clinical trials in humans. Panminerva Med.

[4] Tschöpe C, Ammirati E, Bozkurt B (2021). Myocarditis and inflammatory cardiomyopathy: current evidence and future directions. Nat Rev Cardiol.

[5] Sen-Chowdhry S, Syrris P, Prasad SK (2008). Left-dominant arrhythmogenic cardiomyopathy: an under-recognized clinical entity. J Am Coll Cardiol.

[6] Caforio AL, Calabrese F, Angelini A (2007). A prospective study of biopsy-proven myocarditis: prognostic relevance of clinical and aetiopathogenetic features at diagnosis. Eur Heart J.

[7] Global Burden of Disease Study 2013 Collaborators (2015). Global, regional, and national incidence, prevalence, and years lived with disability for 301 acute and chronic diseases and injuries in 188 countries, 1990-2013: a systematic analysis for the Global Burden of Disease Study 2013. Lancet.

[8] Lota AS, Halliday B, Tayal U (2019). Abstract 11463: epidemiological trends and outcomes of acute myocarditis in the national health service of England. Circulation.

[9] Fazlollahi A, Zahmatyar M, Noori M (2022). Cardiac complications following mRNA COVID-19 vaccines: A systematic review of case reports and case series. Rev Med Virol.

[10] Ling RR, Ramanathan K, Tan FL, Tai BC, Somani J, Fisher D, MacLaren G (2022). Myopericarditis following COVID-19 vaccination and non-COVID-19 vaccination: a systematic review and meta-analysis. Lancet Respir Med.

[11] Montgomery J, Ryan M, Engler R (2021). Myocarditis following immunization with mRNA COVID-19 vaccines in members of the us military. JAMA Cardiol.

[12] Bautista García J, Peña Ortega P, Bonilla Fernández JA, Cárdenes León A, Ramírez Burgos L, Caballero Dorta E (2021). Acute myocarditis after administration of the BNT162b2 vaccine against COVID-19. Rev Esp Cardiol (Engl Ed).

[13] Kim HW, Jenista ER, Wendell DC (2021). Patients with acute myocarditis following mRNA COVID-19 vaccination. JAMA Cardiol.

[14] Shaw KE, Cavalcante JL, Han BK, Gössl M (2021). Possible association between COVID-19 vaccine and myocarditis: clinical and CMR findings. JACC Cardiovasc Imaging.

[15] Jain SS, Steele JM, Fonseca B (2021). COVID-19 vaccination-associated myocarditis in adolescents. Pediatrics.

[16] Truong DT, Dionne A, Muniz JC (2022). Clinically suspected myocarditis temporally related to COVID-19 vaccination in adolescents and young adults: suspected myocarditis after COVID-19 vaccination. Circulation.

[17] D'Angelo T, Cattafi A, Carerj ML (2021). Myocarditis after SARS-CoV-2 vaccination: a vaccine-induced reaction?. Can J Cardiol.

[18] Perez Y, Levy ER, Joshi AY (2021). Myocarditis following COVID-19 mRNA vaccine: a case series and incidence rate determination [IN PRESS]. Clin Infect Dis.

[19] Muthukumar A, Narasimhan M, Li QZ (2021). In-depth evaluation of a case of presumed myocarditis after the second dose of COVID-19 mRNA vaccine. Circulation.

[20] Nevet A (2021). Acute myocarditis associated with anti-COVID-19 vaccination. Clin Exp Vaccine Res.

[21] Naghashzadeh F, Shafaghi S, Dorudinia A (2022). Myocarditis following rAd26 and rAd5 vector-based COVID-19 vaccine: case report. ESC Heart Fail.

[22] Gautam N, Saluja P, Fudim M, Jambhekar K, Pandey T, Al'Aref S (2021). A late presentation of COVID-19 vaccine-induced myocarditis. Cureus.

[23] Parmar K, Mekraksakit P, Del Rio-Pertuz G (2022). Myocarditis following COVID-19 mRNA vaccination. Proc (Bayl Univ Med Cent).

[24] Watkins K, Griffin G, Septaric K, Simon EL (2021). Myocarditis after BNT162b2 vaccination in a healthy male. Am J Emerg Med.

[25] Łaźniak-Pfajfer A, Surmacz R, Rajewska-Tabor J, Pyda M, Lesiak M, Bobkowski W (2022). Myocarditis associated with COVID‑19 vaccination in three male teenagers. Pol Arch Intern Med.

[26] King WW, Petersen MR, Matar RM, Budweg JB, Cuervo Pardo L, Petersen JW (2021). Myocarditis following mRNA vaccination against SARS-CoV-2, a case series. Am Heart J Plus.

[27] Fosch X, Serra J, Torres PL, Preda L, González R, Mojer F (2022). Acute myocarditis after a third dose of the BNT162b2 COVID-19 vaccine. Rev Esp Cardiol (Engl Ed).

[28] Schmitt P, Demoulin R, Poyet R (2021). Acute myocarditis after COVID-19 vaccination: A case report. Rev Med Interne.

[29] Shumkova M, Vassilev D, Karamfiloff K, Ivanova R, Stoyanova K, Yaneva-Sirakova T, Gil RJ (2021). Acute myocarditis associated with the Pfizer/BioNTech vaccine. Kardiol Pol.

[30] Cui G, Li R, Zhao C, Wang DW (2021). Casereport: COVID-19 vaccination associated fulminant myocarditis. Front Cardiovasc Med.

[31] Azir M, Inman B, Webb J, Tannenbaum L (2021). STEMI Mimic: focal myocarditis in an adolescent patient after mRNA COVID-19 Vaccine. J Emerg Med.

[32] Mansour J, Short RG, Bhalla S, Woodard PK, Verma A, Robinson X, Raptis DA (2021). Acute myocarditis after a second dose of the mRNA COVID-19 vaccine: a report of two cases. Clin Imaging.

[33] Riedel PG, Sakai VF, Toniasso SC (2021). Heart failure secondary to myocarditis after SARS-CoV-2 reinfection: a case report. Int J Infect Dis.

[34] Sciaccaluga C, D'Ascenzi F, Cameli M (2022). Case report: two case reports of acute myopericarditis after mRNA COVID-19 vaccine. Front Cardiovasc Med.

[35] Murakami Y, Shinohara M, Oka Y (2022). Myocarditis following a COVID-19 messenger RNA vaccination: a Japanese case series. Intern Med.

[36] Van Kerkhove O, Renders F, Leys M (2022). A case of myocarditis following ChAdOx1 nCov-19 vaccination. Acta Cardiol.

[37] Tailor PD, Feighery AM, El-Sabawi B, Prasad A (2021). Case report: acute myocarditis following the second dose of mRNA-1273 SARS-CoV-2 vaccine. Eur Heart J Case Rep.

[38] Ohnishi M, Tanaka Y, Nishida S, Sugimoto T (2022). Case report of acute myocarditis after administration of coronavirus disease 2019 vaccine in Japan. Eur Heart J Case Rep.

[39] Kawakami T, Yahagi K, Sekiguchi M (2022). Acute myocarditis in a patient following mRNA-1273 SARS-CoV-2 vaccination. Intern Med.

[40] Lee AS, Balakrishnan ID, Khoo CY (2022). Myocarditis following COVID-19 vaccination: a systematic review (October 2020-October 2021). Heart Lung Circ.

[41] Fatima M, Ahmad Cheema H, Ahmed Khan MH (2022). Development of myocarditis and pericarditis after COVID-19 vaccination in adult population: A systematic review. Ann Med Surg (Lond).

[42] Goyal M, Ray I, Mascarenhas D, Kunal S, Sachdeva RA, Ish P (2022). Myocarditis post SARS-CoV-2 vaccination: a systematic review. QJM.

[43] (2022). GMA: Vaccine or virus? CDC says vaccines are still safer for young people than risks of COVID. https://www.goodmorningamerica.com/news/story/vaccine-virus-cdc-vaccines-safer-young-people-risks-78447874.

[44] Blauwet LA, Cooper LT (2010). Myocarditis. Prog Cardiovasc Dis.

[45] Sharma AN, Stultz JR, Bellamkonda N, Amsterdam EA (2019). Fulminant myocarditis: epidemiology, pathogenesis, diagnosis, and management. Am J Cardiol.

[46] Hang W, Chen C, Seubert JM, Wang DW (2020). Fulminant myocarditis: a comprehensive review from etiology to treatments and outcomes. Signal Transduct Target Ther.

[47] Ammirati E, Frigerio M, Adler ED (2020). Management of acute myocarditis and chronic inflammatory cardiomyopathy: an expert consensus document. Circ Heart Fail.

[48] Chen JH, Ikwuanusi IA, Bommu VJ, Patel V, Aujla H, Kaushik V, Cheriyath P (2022). COVID-19 vaccine-related myocarditis: a descriptive study of 40 case reports. Cureus.

[49] Gulin D, Sikic J, Habek JC, Gulin SJ, Galic E (2016). Hypersensitivity eosinophilic myocarditis in a patient receiving multiple drug therapy: challenges in diagnosis and defining the aetiology. Drug Saf Case Rep.

[50] Heymans S, Cooper LT (2022). Myocarditis after COVID-19 mRNA vaccination: clinical observations and potential mechanisms. Nat Rev Cardiol.

[51] McMurray JJ, Adamopoulos S, Anker SD (2012). ESC Guidelines for the diagnosis and treatment of acute and chronic heart failure 2012: The Task Force for the Diagnosis and Treatment of Acute and Chronic Heart Failure 2012 of the European Society of Cardiology. Developed in collaboration with the Heart Failure Association (HFA) of the ESC. Eur Heart J.

[52] Schwitter J, Arai AE (2011). Assessment of cardiac ischaemia and viability: role of cardiovascular magnetic resonance. Eur Heart J.

[53] Bozkurt B, Kamat I, Hotez PJ (2021). Myocarditis With COVID-19 mRNA Vaccines. Circulation.

[54] Haussner W, DeRosa AP, Haussner D, Tran J, Torres-Lavoro J, Kamler J, Shah K (2022). COVID-19 associated myocarditis: A systematic review. Am J Emerg Med.

[55] (2022). EMA: Safety of COVID-19 vaccines. https://www.ema.europa.eu/en/human-regulatory/overview/public-health-threats/coronavirus-disease-covid-19/treatments-vaccines/vaccines-covid-19/safety-covid-19-vaccines.

[56] Matta A, Kunadharaju R, Osman M (2021). Clinical presentation and outcomes of myocarditis post mRNA vaccination: a meta-analysis and systematic review. Cureus.

[57] (2022). CASP Checklists - Critical Appraisal Skills Programme. https://casp-uk.net/casp-tools-checklists/.

